# Vitamin D Ameliorates Skeletal Muscle Atrophy After Spinal Cord Injury by Upregulating TIGAR and Enhancing Mitochondrial Function

**DOI:** 10.1002/jcsm.70401

**Published:** 2026-10-02

**Authors:** Ziyue Dong, Ning Li, Yudong Guo, Haowei Sun, Wenying Shi, Menghai Zhou, Xianming Tong, Zhiyuan Wang, Fangyuan Qian, Yijing Guo

**Affiliations:** ^1^ College of Medicine Southeast University Nanjing China; ^2^ Department of Neurology, Zhongda Hospital Southeast University Nanjing China; ^3^ College of Future Technology The Hong Kong University of Science and Technology (Guangzhou) Guangzhou China; ^4^ Department of Orthopaedics, Zhongda Hospital Southeast University Nanjing China; ^5^ Kangda College Nanjing Medical University Lianyungang China

**Keywords:** energy metabolism, skeletal muscle atrophy, spinal cord injury, vitamin D

## Abstract

**Background:**

Skeletal muscle atrophy following spinal cord injury (SCI) is a debilitating secondary complication with no effective treatment. This study aimed to investigate the therapeutic efficacy of vitamin D (VD) in SCI‐induced muscle atrophy and to elucidate the underlying molecular mechanisms.

**Methods:**

SCI patients' serum VD levels were measured, and muscle mass was assessed using dual‐energy X‐ray examination. Adult male C57BL/6 mice underwent a complete T10 spinal cord transection or sham surgery. On Day 3 post‐injury, mice received daily oral gavage of either VD (150 IU/kg) or vehicle (coconut oil) for 25 days. Body weight and motor function were assessed, and muscle‐specific parameters including hindlimb circumference, muscle mass, fibre cross‐sectional area (CSA) and fibre type composition were evaluated. Quantitative proteomics, Western blotting, and transmission electron microscopy (TEM) were further employed to investigate the molecular mechanisms in the gastrocnemius muscle.

**Results:**

Serum VD level showed a significant positive correlation with the muscle index (*R* ^2^ = 0.6495, *p* < 0.01). Compared to vehicle‐treated SCI mice, VD supplementation significantly attenuated body weight loss and promoted motor recovery (*p* < 0.05). VD treatment markedly preserved hindlimb muscle mass (*p* < 0.01) and prevented the reduction in the CSA of gastrocnemius (*p* < 0.01), extensor digitorum longus (*p* < 0.05) and soleus muscles (*p* < 0.05). Furthermore, VD alleviated the SCI‐induced pathological shift from oxidative (Type I/IIA) to glycolytic (Type IIB) muscle fibres. Proteomic analysis revealed that VD restored cellular energy metabolism homeostasis. And it was related to the upregulation of the metabolic regulator TIGAR (*p* < 0.05). This was associated with a significant increase in mitochondrial number (*p* < 0.001) and an improvement in mitochondrial ultrastructure.

**Conclusion:**

VD mitigates skeletal muscle atrophy following SCI by restoring metabolic and mitochondrial homeostasis through the upregulation of TIGAR. Our findings establish a strong preclinical rationale for investigating VD supplementation as a therapeutic strategy to preserve muscle health in SCI patients.

## 1 Introduction

Spinal cord injury (SCI) represents a devastating neurological condition and a leading cause of permanent disability [1]. A major secondary complication following SCI is a rapid and profound skeletal muscle atrophy below the level of the lesion, driven by neural denervation and mechanical unloading [2]. This debilitating muscle wasting is a primary driver of functional impairment and reduced quality of life. Moreover, such inactivity predisposes patients to a cluster of metabolic disorders, including insulin resistance, type 2 diabetes and cardiovascular disease [3]. Therefore, developing effective therapeutic strategies to counteract muscle atrophy is a critical unmet need in SCI patient care. However, the pathogenesis of skeletal muscle atrophy following SCI is not fully elucidated, and effective therapeutic strategies remain elusive.

Vitamin D (VD), a secosteroid hormone renowned for its classical role in calcium homeostasis and bone metabolism, is increasingly recognised for its extraskeletal functions, particularly in modulating skeletal muscle health [4]. The link between VD and muscle function was initially observed in patients with rickets, who presented with significant muscle weakness [5]. This association is further supported by several clinical studies in aging populations, where lower serum VD levels are predictive of sarcopenia, reduced muscle strength and impaired physical performance [6, 7]. Crucially, a state of VD deficiency is also highly prevalent in the SCI population and has been identified as an independent risk factor for poor motor recovery [8]. However, the intrinsic connection between VD and muscle atrophy remains unknown.

This study first explored the association between VD and muscle atrophy in patients with spinal cord injury. A spinal cord injury mouse model was established to verify the hypothesis that supplementing VD can alleviate skeletal muscle atrophy and improve motor function. To reveal the underlying molecular mechanisms, we employed quantitative proteomics methods to investigate the changes in cellular energy metabolism and mitochondrial homeostasis caused by VD.

## 2 Materials and Methods

### 2.1 Participants

This study included 12 patients with traumatic spinal cord injury who visited the Zhongda Hospital of Southeast University from April 2024 to April 2025. The inclusion criteria were injury at the cervical or thoracic spinal cord level, having undergone surgical treatment, ASIA classification as B‐D, age ranging from 18 to 75 years, and a disease duration of more than 6 months. The exclusion criteria included combined fractures of the limbs, other neuromuscular diseases, malignant tumours, end‐stage chronic kidney disease, severe mental disorders, X‐ray contraindications (such as severe claustrophobia, etc.), or those who could not cooperate with the examinations. All participants signed the informed consent form, and this study was approved by the Ethics Committee of Zhongda Hospital of Southeast University (2024ZDSYLL111‐P01).

### 2.2 Clinical Assessments

The researchers collected the clinical data of the patients, including their gender, age, disease duration, injury level, and previous medical history, and evaluated the severity of the injury. The patients were scanned throughout their bodies using a dual‐energy X‐ray densitometer (Horizon, Hologic, Marlborough, Massachusetts, United States), and the appendicular skeletal muscle mass (ASM) of the patients was obtained. The ASM was then adjusted to the square of height to calculate the muscle index (expressed in kg/m^2^). This was carried out by a specialist physician and reviewed by another specialist.

### 2.3 Animals and Ethical Approval

All animal procedures were performed following the guidelines approved by the Experimental Animal Ethics Committee of Southeast University. Eight‐week‐old male C57BL/6 mice were used and group‐housed at the Laboratory Animal Center under specific pathogen‐free conditions on a 12‐h light/dark cycle at a controlled temperature, with ad libitum access to standard chow and water.

### 2.4 SCI Model Establishment and Treatments

The study was designed as follows (Figure 1b). Mice were randomly assigned to 4 groups (*n* = 6): sham‐operated + vehicle (SHAM‐OIL), sham‐operated + VD treatment (SHAM‐VD), SCI + vehicle (SCI‐OIL) and SCI + VD (SCI‐VD). Anaesthesia was induced with 4% and maintained with 1%–2% isoflurane, respectively. Following dorsal shaving and sterilisation of the surgical site with povidone‐iodine, a midline incision was made to expose the vertebral column. A laminectomy was performed at the T10 level. For the SCI groups, the spinal cord was completely transected using microscissors. For the sham groups, only the laminectomy was performed. Following the procedure, the muscle layers and skin were sutured. Mice were placed on a heating pad until fully recovered from anaesthesia. Successful SCI was confirmed by the complete paralysis of both hindlimbs (BMS score of 0) 24 h post‐surgery. Postoperatively, all mice were housed in pairs with ad libitum access to standard chow and water. Manual bladder expression was performed *b.i.d*. until the recovery of urinary function.

**FIGURE 1:**
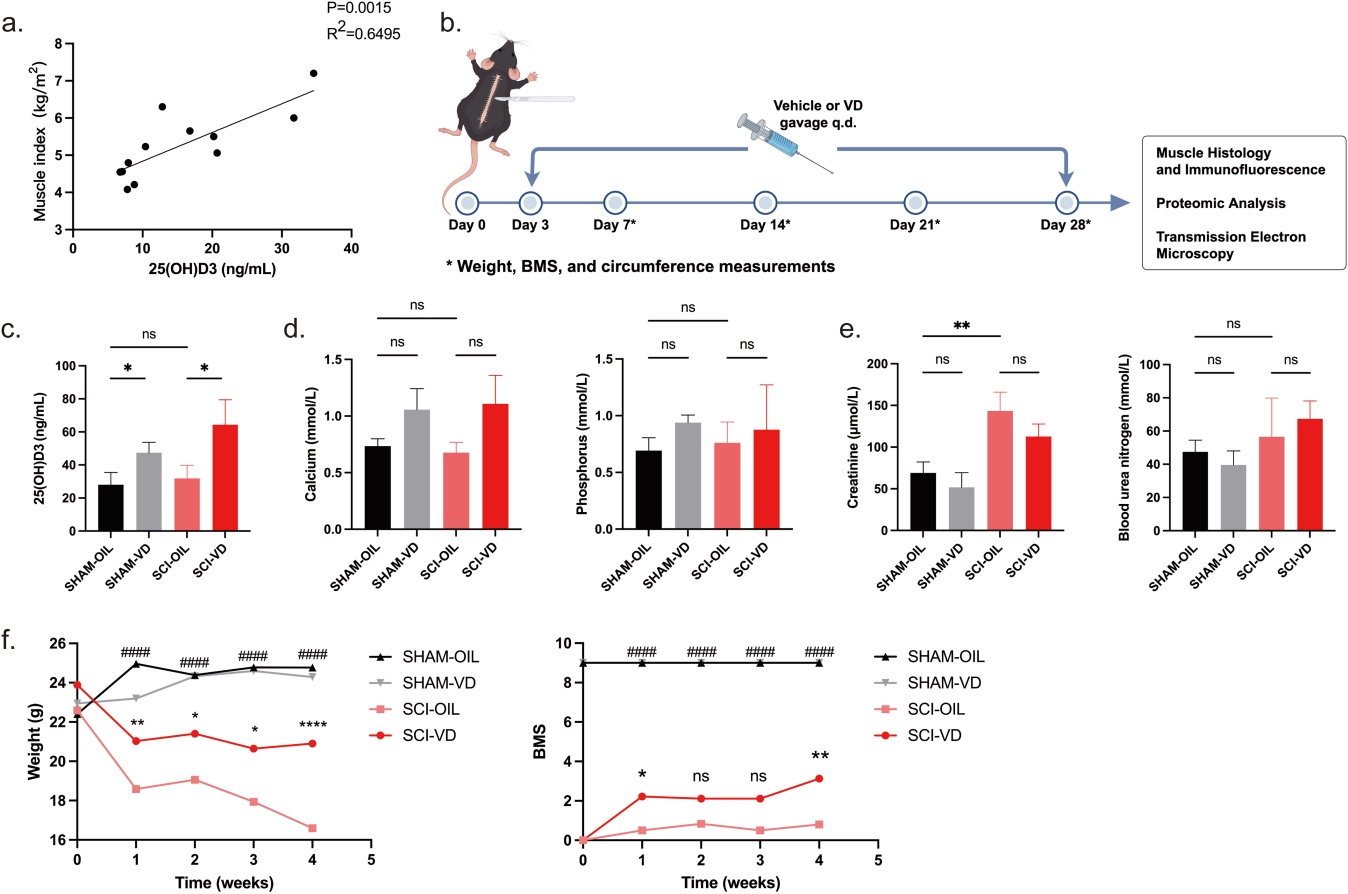
Vitamin D attenuates body weight loss and promotes motor function recovery following SCI. (a) Serum VD level showed a significant negative correlation with the muscle index. (b) The body weight, BMS and hind limb circumference were assessed on the 7th, 14th, 21st and 28th days after the operation, and the remaining work was performed after the 28th day. (c–e) VD treatment significantly increases serum 25(OH)D3 levels without causing significant increases in calcium, phosphate, creatinine or urea nitrogen. (f) Left: Weekly body weight measurements. SCI induced a sustained loss of body weight, which was significantly attenuated by VD supplementation. Right: Weekly assessment of hindlimb motor function using the BMS. VD treatment led to a significant improvement in BMS scores compared to the vehicle‐treated SCI group at Weeks 1 and 4. *p < 0.05, **p < 0.01, ****p < 0.0001, SCI‐VD vs. SCI‐OIL. ^####^ *p* < 0.0001, SCI‐VD vs. SHAM‐OIL.

Beginning on Day 3 post‐injury, mice in the VD groups received a daily oral gavage of VD (150 IU/kg, Calcitriol, Catalent Germany) dissolved in coconut oil [9]. The OIL groups received an equivalent volume of the vehicle. Treatments continued for 25 consecutive days.

### 2.5 Functional and Morphometric Assessments

Body weight, hindlimb circumference, and motor function were evaluated weekly on Days 7, 14, 21 and 28. Motor function of the hindlimbs was assessed in an open field for 2 min per mouse using the Basso Mouse Scale (BMS) for locomotion. BMS score assesses the joint movement, supporting force and gait coordination of the hind limbs of mice. Zero point indicates no joint movement (hip, knee, ankle), 1 and 2 represent slight and significant joint movement respectively, 3 indicates coordinated joint movement, 4 and above represent the hind limbs can support the body and the continuity and coordination of the gait are gradually evaluated, and 9 indicates a completely normal gait [10]. For body weight and circumference measurements, mice were briefly anaesthetised with 2.5% isoflurane to minimise stress. Hindlimb circumference was measured at the midpoint between the knee and ankle joint. All experiment was performed by two independent investigators in a blinded manner, and the average score was used for further analysis.

### 2.6 Assessment of Serum 25(OH)D3, Calcium, Phosphate, Creatinine and Urea Nitrogen Levels

Patients' blood samples were collected from the antecubital veins. Mice's blood samples were collected from the posterior orbital vein of the anaesthetised mice. Whole blood was allowed to clot at room temperature for 30 min. Serum was separated by centrifugation at 3000 rpm for 10 min at 4 °C. Patients' serum 25(OH)D3 level was determined using a fully automated biochemical analyser (Beckman, Germany). Mice's 25(OH)D3 level was detected using 25(OH)D3 ELISA kit (Elabscience); calcium, phosphate, creatinine and urea nitrogen levels were detected using assay kits (both Jiancheng) according to the manufacturer's instructions.

### 2.7 Muscle Tissue Collection and Processing

At the endpoint of day 28, mice were euthanised by anaesthetic overdose followed by cervical dislocation. The tibialis anterior (TA), extensor digitorum longus (EDL), gastrocnemius (GA) and soleus (SOL) muscles were rapidly dissected from both hindlimbs, and adherent connective tissue was carefully removed. Each muscle was blotted dry, and its wet weight was recorded. Subsequently, muscles for histological and immunofluorescence analyses were mounted on cork in gum tragacanth, snap‐frozen in isopentane pre‐chilled with liquid nitrogen and stored at −80 °C. For proteomic and Western blotting analyses, muscles were immediately flash‐frozen in liquid nitrogen and stored at −80 °C for further use. For transmission electron microscopy (TEM), small portions of the GA muscle were cut into 1 mm^3^ blocks and fixed in 2.5% glutaraldehyde in 0.1 M cacodylate buffer (pH 7.4) at 4 °C for further procedures.

### 2.8 Muscle Histology and Immunofluorescence

To assess muscle fibre morphology, 8 μm transverse cryosections of the GA, TA, EDL and SOL muscle were stained with haematoxylin and eosin (H&E) using standard protocol. Briefly, images were taken using microscope (U‐25ND25, Olympus). Muscle fibre cross‐sectional area (CSA) was quantified using ImageJ software 1.8.0 in a blinded manner.

For immunofluorescence (IF), 10 μm sections were fixed in ice‐cold acetone, permeabilised with 0.1% Triton X‐100 (biofroxx) and blocked with 5% bovine serum albumin (Biofroxx). Sections were then incubated overnight at 4 °C with primary antibodies targeting different myosin heavy chain (MyHC) isoforms to identify fibre types (I: 1:500, BA‐D5, DSHB) (IIA: 1:200, SC‐71, DSHB) (IIB: 1:50, BF‐F3, DSHB). After washing, sections were incubated with corresponding Alexa Fluor‐conjugated secondary antibodies (1:500, A‐21042, A‐21124, A‐21242, Thermo Fisher) and counterstained with DAPI (Beyotime). All slices were kept from light at 4 °C. Images were captured using a digital scanner (VS200, Olympus).

### 2.9 Proteomic and Bioinformatic Analysis

For quantitative proteomic analysis, total protein was extracted from GA muscle tissue (*n* = 4) using RIPA buffer supplemented with a protease inhibitor cocktail. Following quantification with a BCA assay, 50 μg of protein per sample was reduced with dithiothreitol, alkylated with iodoacetamide and digested overnight with sequencing‐grade trypsin. The resulting peptides were desalted using C18 StageTips and analysed via nano‐liquid chromatography–tandem mass spectrometry (nLC‐MS/MS) on a Q Exactive HF‐X Hybrid Quadrupole‐Orbitrap mass spectrometer coupled to an EASY‐nLC 1200 system (Thermo Fisher Scientific, United States). Peptides were separated on an Acclaim PepMap C18 analytical column over a 120‐min linear gradient and the mass spectrometer was operated in data‐dependent acquisition (DDA) mode. Raw data files were processed using MaxQuant software (v1.6.17.0) for label‐free quantification (LFQ) against the UniProt *Mus musculus* database, with a false discovery rate (FDR) set to < 1%. Differentially expressed proteins (DEPs) were identified using a two‐sample *t*‐test, applying significance thresholds of *p* value < 0.05 and an absolute log2(Fold Change) ≥ 1.5. Functional enrichment analysis for Gene Ontology (GO) terms and Kyoto Encyclopedia of Genes and Genomes (KEGG) pathways was performed using the DAVID bioinformatics resource (v6.8).

### 2.10 Western Blotting

Total protein was extracted from GA muscle tissue using RIPA buffer supplemented with protease and phosphatase inhibitors. Protein concentration was determined using a BCA assay kit. Equal amounts of protein (20 μg) per sample were separated by SDS‐PAGE and transferred to a PVDF membrane. Membranes were blocked in 5% BSA in TBST and incubated overnight at 4 °C with the following primary antibodies: anti‐TIGAR (1:1000, 22136‐1‐AP, Proteintech), anti‐FBP2 (1:1000, 25192‐1‐AP, Proteintech) and GAPDH (1:50000, 60004‐1‐Ig, Proteintech). After incubation with HRP‐conjugated secondary antibodies (1:10000, SA00001‐1 and SA00001‐2, Proteintech), protein bands were visualised using an ECL detection kit (Life‐iLab). Band intensities were quantified using ImageJ.

### 2.11 Transmission Electron Microscopy

For ultrastructural analysis, fixed GA muscle chunks were then post‐fixed with 1% osmium tetroxide, dehydrated through a graded ethanol series and embedded in Epon resin. Ultrathin sections (~90 nm) were cut using a diamond knife, mounted on copper grids, and stained with uranyl acetate and lead citrate. The sections were examined and imaged using a Thermo Fisher Talos F200X transmission electron microscope. For quantitative analysis, images of non‐overlapping, transversely sectioned muscle fibres were captured at 7000× magnification. The number of mitochondria per unit area was then quantified from at least five random fields per animal by an investigator blinded to the treatment groups.

### 2.12 Statistical Analysis

Statistical analyses were performed using GraphPad Prism 10.2.0 (GraphPad Software Inc., United States). All data are presented as the mean ± standard deviation (SD). Normality of data distribution was assessed using the Shapiro–Wilk test. Longitudinal data measured over time (body weight, BMS score and hindlimb circumference) were analysed using a two‐way repeated measures ANOVA followed by Bonferroni's post hoc test for multiple comparisons. For single time‐point data collected at the experimental endpoint (serum indicators, muscle mass, muscle fibre cross‐sectional area, fibre type percentages, Western blot quantification and mitochondrial number), differences were analysed by one‐way ANOVA followed by Tukey's post hoc test. The correlation analysis between continuous variables was analysed by Pearson correlation (for normal distribution) or Spearman correlation (for non‐normal distribution). A *p* value of less than 0.05 was considered statistically significant.

## 3 Results

### 3.1 Serum VD Levels Exhibit a Significant Correlation With the Severity of Muscle Atrophy in Chronic SCI Patients

Twelve patients with chronic SCI participated in this study. Their baseline characteristics, including disease duration, injury severity, serum VD levels and muscle indices, are summarised in Table 1. A majority of the patients (8/12) had deficient serum VD levels (< 20 ng/mL). Correlation analysis revealed a significant positive association between serum VD levels and muscle indices (*R* ^2^ = 0.6495, *p* < 0.01, Figure 1a), indicating a concurrent significant inverse correlation with the degree of skeletal muscle atrophy.

**TABLE 1:** Participant characteristics.

Patient	1	2	3	4	5	6	7	8	9	10	11	12
Age (years)	60	25	60	37	54	67	28	24	65	42	71	67
Gender (F/M)	F	M	M	F	F	M	M	M	M	F	F	M
Time postinjury (m)	9	30	24	18	23	16	25	10	32	11	14	22
Injury level	T10	C8	C7	T8	C5	C4	C4	C5–7	C4	T11	T11	T6
AISA	D	C	B	C	C	C	B	B	B	D	D	C
25(OH)D3 (ng/mL)	7.82	8.8	6.77	7.08	7.94	31.72	10.41	20.22	12.83	16.78	20.69	34.56
Muscle index (kg/m^2^)	4.08	4.21	4.55	4.56	4.8	6	5.23	5.5	6.3	5.65	5.6	7.2

### 3.2 VD Treatment Increases Serum VD Levels in SCI Mice

To determine whether this clinical correlation translates into a viable therapeutic strategy, we transitioned to an *in vivo* SCI mouse model. The safety and systemic elevation of serum VD levels following oral supplementation were verified prior to evaluating its efficacy. Serum 25(OH)D3 levels were significantly elevated in VD treated mice compared to the vehicle group (*p* < 0.05, Figure 1c). To monitor the potential side effects of VD, we also measured serum calcium, phosphate, creatinine, and urea nitrogen levels. VD treated mice showed slight increases in serum calcium and phosphorus compared to the placebo group; these changes did not reach statistical significance (*p* > 0.05, Figure 1d) and remained within the normal physiological range. After SCI, creatinine levels were significantly elevated (*p* < 0.01, Figure 1e). VD treatment did not induce significant changes in either creatinine or urea nitrogen levels (*p* > 0.05, Figure 1e), indicating that the administered VD dosage had no adverse effects on renal function. We suppose that the elevated creatinine levels may primarily stem from neurogenic muscle injury leading to increased creatinine production, rather than renal impairment. This inference is drawn from the concurrent observation that urea nitrogen levels remained unchanged, a pattern more indicative of a muscular rather than a renal origin, even though urinary retention following spinal cord injury may potentially contribute to renal damage.

### 3.3 VD Relieves the Severity of Body Weight and Motor Function Loss Following SCI

To evaluate the systemic effects of VD following SCI, we monitored body weight and hindlimb motor function weekly for 28 days. As expected, SCI induced a profound and sustained loss of body weight in vehicle‐treated mice (SCI‐OIL) compared to sham controls (SHAM‐OIL) throughout the experimental period (*p* < 0.0001). Notably, daily VD supplementation significantly attenuated this weight loss, with the SCI‐VD group maintaining a higher body weight than the SCI‐OIL group at all time points, reaching high significance by week 4 (*p* < 0.0001) (Figure 1f). Concurrently, motor function, assessed by the BMS, was severely impaired after SCI, and the SCI‐OIL group showed minimal spontaneous recovery. In contrast, VD treatment led to a significant improvement in BMS scores compared to the vehicle group at Week 1 (*p* < 0.05) and week 4 (*p* < 0.01) (Figure 1f), but still less than the SHAM‐OIL group, indicating that VD minimises the severity of the motor function loss slightly.

### 3.4 VD Mitigates Skeletal Muscle Atrophy and Preserves Muscle Fibre Size After SCI

We next assessed the direct impact of VD on skeletal muscle wasting. At 28 days post‐injury, qualitative examination revealed marked atrophy in the TA, EDL, GA and SOL muscles of SCI‐OIL mice, an effect that was visibly attenuated in the SCI‐VD group (Figure 2a). This was further evidenced by hindlimb circumference measurements, which were significantly greater in the SCI‐VD group than the SCI‐OIL group at weeks 2 and 4 (Figure 2b). SCI led to a significant reduction in the mass of the GA muscle. After standardising the muscle mass with body weight, this change remained meaningful, and this reduction was partially but significantly alleviated by VD treatment (*p* < 0.01, Figure 2c).

**FIGURE 2:**
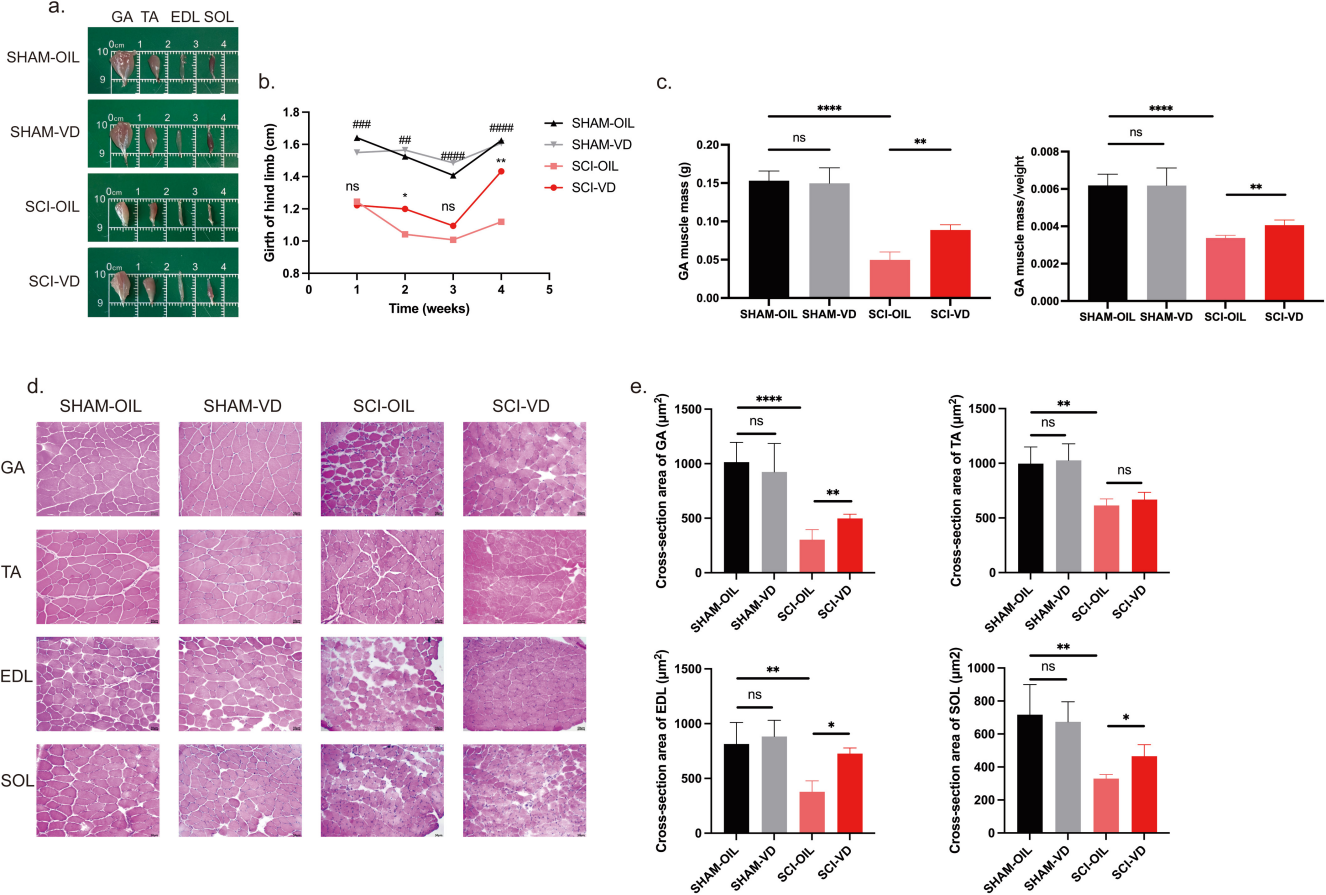
VD mitigates denervation‐induced skeletal muscle atrophy. (a) Representative images of dissected hindlimb muscles at 28 days post‐injury, showing that VD treatment visibly attenuated muscle atrophy. (b) Weekly hindlimb circumference measurements, which were significantly greater in the SCI‐VD group (* SCI‐OIL vs. SCI‐VD; # SCI‐OIL vs. SHAM‐OIL). (c) VD treatment significantly alleviated the decline in muscle mass after SCI. (d) Representative H&E‐stained transverse sections. (e) Quantification of mean muscle fibre CSA. VD treatment preserved a more normal muscle fibre morphology, and significantly increased the CSA in GA, EDL and SOL muscles. *p < 0.05, **/^##^ *p* < 0.01, ^###^ *p* < 0.001, ****/^####^ *p* < 0.0001. Magnification: 200.

To investigate the cellular basis for this muscle preservation, we performed histological analysis. H&E staining showed that the well‐organised, polygonal muscle fibres seen in sham mice were replaced by smaller, irregularly shaped fibres with increased interstitial space in the SCI‐OIL group. VD treatment largely preserved the muscle fibre morphology (Figure 2d). Quantification of the CSA confirmed these observations. SCI induced a significant reduction in fibre CSA across all examined muscles. Importantly, VD treatment resulted in a significant increase in the mean CSA of GA, EDL and SOL muscles compared to the vehicle‐treated SCI group (Figure 2e). No statistically significant increase in CSA was observed in the TA muscle (Figure 2e).

### 3.5 VD Alleviates the SCI‐Induced Shift From Oxidative to Glycolytic Muscle Fibre Types

Given that muscle atrophy is often accompanied by changes in fibre type composition, we performed immunofluorescence staining for different MyHC isoforms. Following SCI, we observed a significant shift away from oxidative fibres. In the GA muscles, the proportions of Type I and IIA muscle fibres in the SCI‐OIL group were significantly reduced (I, *p* < 0.01; IIA, *p* < 0.001), the proportion of Type IIB muscle fibres increased significantly (*p* < 0.01). These changes were alleviated by VD treatment (I, *p* < 0.01; IIA, *p* < 0.01; IIB, *p* < 0.05) (Figure 3a,b). In the TA muscles, Type I muscle fibres were not expressed and not included in the statistics. The proportion of Type IIA muscle fibres decreased significantly after SCI (*p* < 0.001) and increased significantly after VD treatment (*p* < 0.01), while there was no significant change in Type IIB muscle fibres (Figure 3a,c). In the EDL muscles, Type I muscle fibres were not expressed, and the proportion of Type IIA muscle fibres decreased after SCI but did not reach statistical significance, and significantly decreased after VD treatment (*p* < 0.05), while there was no significant change in Type IIB muscle fibres (Figure 3a,d). The most striking change occurred in the SOL muscle, a predominantly slow‐twitch muscle, which underwent a dramatic transformation towards fast‐twitch, glycolytic Type IIB fibres after SCI. Remarkably, VD treatment almost completely prevented this undesirable fibre type conversion, resulting in a significant decrease in the proportion of IIB fibres (*p* < 0.0001) (Figure 3a,e).

**FIGURE 3:**
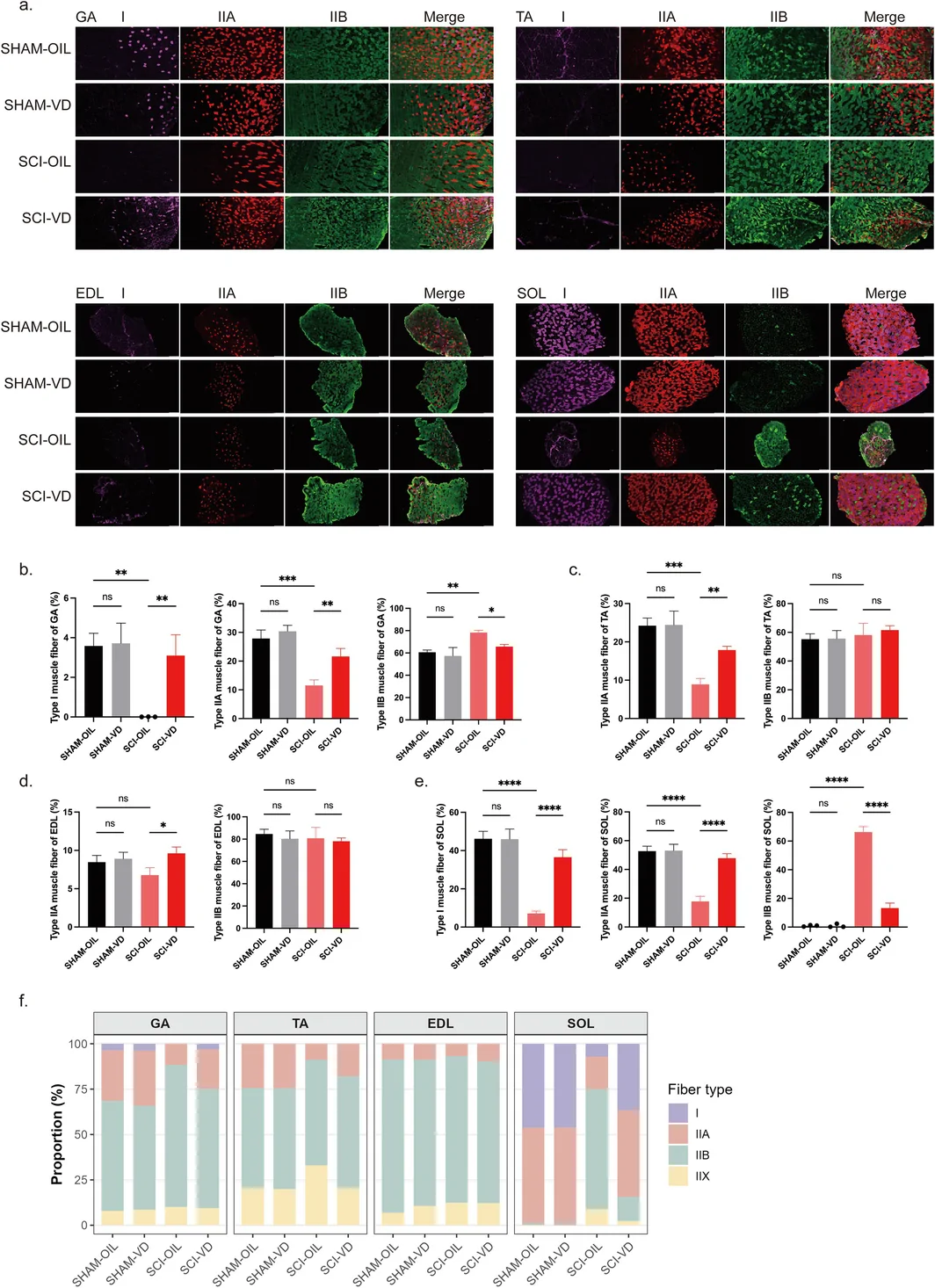
VD alleviates the SCI‐induced shift from an oxidative to a glycolytic muscle fibre phenotype. (a) Immunofluorescence images of GA, TA, EDL and SOL muscles. (b) Muscle fibre proportion of GA. Type I and IIa muscle fibres decreased significantly after SCI and increased significantly after VD treatment. Type IIB muscle fibres increased significantly after SCI and decreased after VD treatment. (c) Muscle fibre proportion of TA. Type IIA muscle fibres significantly decreased after SCI and significantly increased after VD treatment. Type IIB muscle fibres showed no significant change. (d) Muscle fibre proportion of EDL. Type IIA muscle fibres showed no significant change after SCI but significantly increased after VD treatment. Type IIB muscle fibres showed no significant change. (e) Muscle fibre proportion of SOL. Type I and IIa muscle fibres decreased significantly after SCI and increased significantly after VD treatment. Type IIB muscle fibres increased significantly after SCI and decreased after VD treatment. (f) Stacked bar graph of muscle fibre proportion. *p < 0.05, **p < 0.01, ***p < 0.001, ****p < 0.0001. Magnification: 50.

### 3.6 VD May Restores Metabolic and Mitochondrial Homeostasis in Muscles After SCI

To elucidate the molecular mechanisms underlying VD's protective effects, we performed quantitative proteomic analysis on GA muscles. Unsupervised hierarchical clustering clearly separated the four experimental groups (Figure 4a). Principal component analysis (PCA) revealed that SCI caused severe and global changes in protein expression. The protein expressions in the SCI‐OIL and SCI‐VD groups showed some overlap, suggesting that VD treatment had a relatively mild effect on some proteins. Although the therapeutic effect of VD was mild, when the sham group was included in the comparison, we found that the protein expression signature of the VD treatment group was closer to that of the sham group. This suggests that VD treatment causes the overall protein profile to shift towards a normal direction (Figure 4b). We identified 171 proteins that were altered by SCI and subsequently restored towards sham levels by VD treatment (Figure 4c). Gene Ontology (GO) and KEGG pathway enrichment analysis of these 171 proteins revealed a significant enrichment in pathways related to cellular metabolism, including glycolysis/gluconeogenesis (Figure 4d).

**FIGURE 4:**
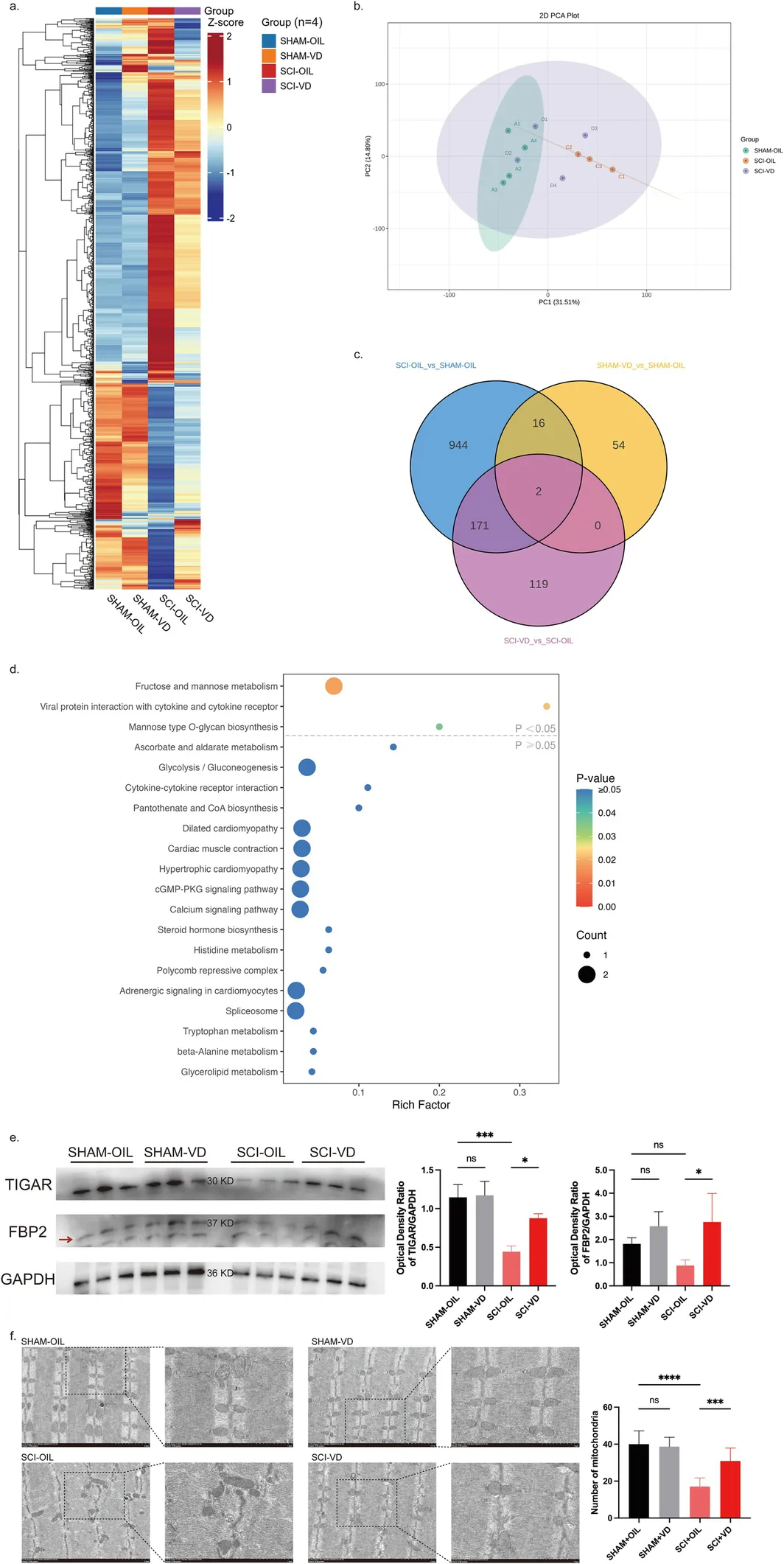
Proteomics suggested that VD restored metabolic and mitochondrial homeostasis in atrophied muscle. (a,b) Both the hierarchical clustering heatmap and the PCA demonstrated clear separation between SHAM and SCI groups along PC1, with partial overlap between SCI‐OIL and SCI‐VD; the SCI‐VD cluster was shifted towards SHAM relative to SCI‐OIL. (c) Venn diagram identified 171 proteins that were altered by SCI and subsequently restored towards sham levels by VD treatment. (d) KEGG pathway analysis of these proteins showed a significant enrichment in pathways related to cellular metabolism, including glycolysis/gluconeogenesis. (e) Immunoblotting demonstrated that VD treatment significantly upregulated the expression of both TIGAR and FBP2 compared to vehicle‐treated SCI muscle. (f) Representative TEM images revealed that SCI caused a significant loss of mitochondria and disorganisation of their cristae. VD treatment markedly improved mitochondrial morphology and significantly restored mitochondrial density. *p < 0.05, **p < 0.01, ***p < 0.001, ****p < 0.0001. Magnification: 7.0k and 15k.

Based on this analysis, we selected two highly regulated proteins in these pathways, TIGAR and FBP2, for validation by Western blotting. The proteomic analysis results showed that the protein expression of Tigar after SCI was only 44% of that in the SHAM‐OIL group, and the expression of Fbp2 decreased to 39% of that in the SHAM‐OIL group. After VD treatment, the expression of Tigar in the SCI‐VD group showed a 1.68‐fold increase compared to the SCI‐OIL group, and the expression of Fbp2 was 1.79‐fold higher. The Western blotting analysis confirmed that TIGAR expression was significantly downregulated in the SCI‐OIL group and robustly upregulated by VD treatment (*p* < 0.05). Similarly, FBP2 expression was significantly increased in the SCI‐VD group compared to the SCI‐OIL group (*p* < 0.05) (Figure 4e). Given that TIGAR is a known regulator of mitochondrial function and biogenesis, we next examined mitochondrial ultrastructure using TEM. We selected the comparable fields of view in different sections for comparison. The position arrangement of mitochondria has undergone significant changes. In the sham groups, mitochondria were evenly distributed on both sides of the Z‐line. After spinal cord injury, the Z‐line broke down and the mitochondrial arrangement became disordered. In the sham groups, the number of mitochondria was abundant. In contrast, the number of mitochondria in the muscles of SCI‐OIL mice was significantly reduced. VD treatment improved the morphology and arrangement of mitochondria and significantly restored the number of mitochondria in each muscle fibre region (*p* < 0.001) (Figure 4f). These results suggest that VD mitigates muscle atrophy by improving cellular energy metabolism and promoting mitochondrial health.

## 4 Discussion

The clinical study reveals a direct correlation between serum VD concentration and the extent of muscle atrophy in SCI population, suggesting the potential therapeutic role of VD in treating muscle atrophy after SCI. Subsequent research further substantiates that VD supplementation represents a viable therapeutic strategy to mitigate skeletal muscle wasting following severe SCI. Our findings demonstrate that in a mouse model of complete spinal cord transection, VD administration not only attenuated body weight loss and promoted motor function recovery partially, but also fundamentally preserved skeletal muscle mass and fibre architecture. At the molecular level, we have uncovered a mechanism whereby VD counteracts the maladaptive shift from oxidative to glycolytic muscle fibre types. This could be mediated by the restoration of metabolic homeostasis through the upregulation of the key metabolic regulator TIGAR and FBP2, and the subsequent enhancement of mitochondrial quantity and quality.

Skeletal muscle is a highly plastic and metabolically dynamic tissue, with its functional capacity being largely dictated by the composition of distinct myofiber types. These fibres are broadly classified based on their bioenergetic profiles and contractile properties. At one end of the spectrum are the Type I, or slow‐twitch oxidative, fibres. Characterised by high mitochondrial density, rich myoglobin content and extensive capillarisation, they are specialised for sustained, low‐intensity contractions and are paramount for endurance and postural control. At the opposite end are the Type IIB, or fast‐twitch glycolytic, fibres, which rely predominantly on anaerobic glycolysis for rapid ATP generation. They are optimised for powerful, short‐duration efforts but are consequently more susceptible to fatigue. Type IIA fibres exhibit intermediate properties and possess remarkable plasticity, allowing the muscle to adapt to varying functional demands. The homeostatic balance among these fibre types is critical for overall muscle health, and its disruption is a central feature of muscle wasting [11, 12, 13]. For instance, in conditions of disuse such as prolonged immobilisation, skeletal muscle undergoes significant atrophy characterised by a preferential loss of slow‐twitch fibres and a general shift towards a faster, more glycolytic phenotype [14, 15]. This pathological remodelling is amplified following complete denervation due to spinal cord injury, where a near‐total transformation to a muscle composed almost exclusively of fast, highly fatigable fibres becomes a definitive pathological hallmark.

Aligning with this pathological framework, our study shows that SCI induces a profound and detrimental shift in this balance. We observed the overall trend of the muscle transformation after SCI is from fatigue‐resistant, oxidative fibres (Type I and IIA) to fatigue‐prone, glycolytic Type IIB fibres, a response particularly pronounced in GA and SOL muscles. Although this characteristic was not fully manifested in some muscles, for instance, the TA muscle showed only a reduction in type IIA muscle fibres, and the EDL showed no significant change. This conversion represents a documented hallmark of neuromuscular disconnection, leading to compromised metabolic efficiency and exacerbating the cycle of muscle wasting. The more striking finding of our work is the efficacy of VD in preventing this maladaptive fibre type switch. This suggests that VD's protective effects extend far beyond simply preserving muscle mass; it may actively safeguard the fundamental functional and metabolic identity of the muscle tissue. By preserving a more oxidative phenotype, VD may help counteract the systemic metabolic dysregulation.

The maintenance of skeletal muscle mass and function is intrinsically linked to its bioenergetic state, with mitochondria serving as the central hub for energy production and metabolic regulation. Consequently, mitochondrial dysfunction and impaired energy metabolism are now recognised as core pathological features across a wide spectrum of muscle wasting conditions [16, 17]. Our work aligns perfectly with this paradigm. The quantitative proteomic analysis of muscles from SCI mice revealed that pathways related to cellular energy metabolism were among the most significantly dysregulated. This finding strongly suggests that restoring metabolic homeostasis is a critical prerequisite for counteracting the severe muscle atrophy that follows denervation.

Further interrogation of our proteomic data led us to identify TIGAR and FBP2 as prime candidates for mediating its protective effects. Both proteins are pivotal regulators of glucose metabolism. TIGAR, in particular, has emerged as a multifunctional protein with specialised roles in skeletal muscle. It not only suppresses excessive glycolysis but also shunts glucose flux into the pentose phosphate pathway, thereby boosting the production of NADPH, which is essential for combating oxidative stress [18]. Crucially, recent studies have demonstrated that TIGAR is highly expressed in skeletal muscle and is indispensable for maintaining mitochondrial function and enhancing exercise endurance, partly through the activation of the SIRT1‐PGC‐1α signalling axis [19, 20]. FBP2, the muscle‐specific isoform of fructose‐1,6‐bisphosphatase, also possesses non‐canonical functions beyond gluconeogenesis, including the direct protection of mitochondria from stress‐induced damage [21]. Therefore, upregulating these proteins, especially TIGAR, could serve as a potent strategy for preserving muscle health. In complete agreement with this hypothesis, our Western blot analysis confirmed that VD treatment robustly upregulated both TIGAR and FBP2 expression in the atrophied muscles of SCI mice. This molecular restoration was functionally correlated with a marked increase in mitochondrial number and a significant improvement in mitochondrial ultrastructure, as evidenced by our TEM analysis.

VD is increasingly recognised for its extraskeletal functions, including the modulation of cellular energy metabolism [22, 23]. Current research indicates that the VD receptor exists in various cells, including skeletal muscle cells [24]. Girgis et al. discovered through immunofluorescence staining that VDR is mainly located in the cytoplasm and nucleus of muscle fibres [25]. Ryan et al. confirmed that VDR exists in the cytoplasm of human skeletal muscle cells, and that VD can regulate the physiological functions, morphology and expression of key mitochondrial proteins of muscle cells, thereby affecting muscle strength [26]. Sinha et al. evaluated the mitochondrial oxidative phosphorylation function in skeletal muscle using phosphorus‐31 magnetic resonance spectroscopy. They found that after VD supplementation, the rate of mitochondrial oxidative phosphorylation improved, demonstrating a direct link between VD status and the function of skeletal muscle mitochondria [27]. Salles et al. observed that in vivo and in vitro VD fluctuations changed mitochondrial biogenesis and oxidative activity in skeletal muscle [28]. In other cell types, VD deficiency has been linked to reduced mitochondrial activity and increased oxidative stress, whereas supplementation can improve mitochondrial function. Specifically, VD has been shown to regulate key enzymes involved in glucose metabolism and enhance oxidative phosphorylation capacity [29, 30]. Integrating these established roles with our novel findings, we propose that VD exerts its protective effects on denervated muscle by orchestrating a profound metabolic reprogramming through upregulation of TIGAR. This restoration of metabolic and mitochondrial homeostasis is likely the fundamental mechanism that underpins the preservation of the oxidative muscle fibre phenotype and the ultimate mitigation of SCI‐induced muscle atrophy.

While this study provides robust preclinical evidence for the benefits of VD, we recognise several avenues for future investigation that will build upon our findings. The primary next step is to definitively establish the causal role of the TIGAR pathway in mediating VD's protective effects. Although our data strongly implicates TIGAR upregulation as a central mechanism, future studies employing genetic models, such as muscle‐specific TIGAR knockout mice, will be crucial to determine if this pathway is indispensable. Furthermore, to provide a more comprehensive physiological assessment, future work should complement our morphological findings with direct functional evaluations of muscle contractility and fatigue resistance. Such studies will not only solidify the mechanistic basis but also strengthen the rationale for translating these findings into clinical strategies for SCI patients.

## 5 Conclusions

In conclusion, our study elucidates a critical, previously unrecognised role for vitamin D in preserving skeletal muscle integrity following severe spinal cord injury. We demonstrate that VD's therapeutic benefits are mediated by a profound metabolic reprogramming within the denervated muscle. Specifically, VD upregulates the key metabolic regulator TIGAR, which in turn restores mitochondrial homeostasis and alleviates the pathological shift towards a glycolytic fibre type phenotype. These findings identify the VD‐TIGAR‐mitochondrial axis as a novel and promising therapeutic target to combat muscle wasting and improve functional outcomes for individuals living with SCI.

## Funding

This work was supported by Natural Science Foundation of Jiangsu Province (Grant No. BK20242099 to Ning Li), Zhongda Hospital Affiliated to Southeast University, Jiangsu Province High‐Level Hospital Construction Funds (GSP‐LCYJFH25) and The Healthy China Fund of the China Social Work Development Foundation (HIGHER2022120).

## Conflicts of Interest

The authors declare no conflicts of interest.
